# Anticipating Environmental Burdens in Research and Innovation Projects—Application to the Case of Active and Healthy Ageing

**DOI:** 10.3390/ijerph17103600

**Published:** 2020-05-20

**Authors:** Irene Monsonís-Payá, Tomás Gómez-Navarro, Mónica García-Melón

**Affiliations:** 1 INGENIO (CSIC-UPV), Universitat Politècnica de València, Cámino de Vera s/n, 46022 Valènci, Spain; irmonpa@doctor.upv.es (I.M.-P.); mgarciam@dpi.upv.es (M.G.-M.); 2 Institute of Energy Engineering IIE, Universitat Politècnica de València, 46022 Valenci, Spain

**Keywords:** responsible innovation, environmental sustainability, ICT and active and healthy aging, AHP

## Abstract

In this paper; for research and innovation projects without environmental goals; a procedure is proposed to operationalize the anticipation and reflexivity of environmental concerns in the initial phases. By using the expert knowledge of specialists; we have first conducted a study to identify the general environmental topics relevant in any kind of research and innovation project not addressing the environment. In a second phase; a strategy is proposed to rank order the topics in terms of environmental relevance by means of the Analytic Hierarchy Process. To illustrate it; the case of Information and Communication Technologies for Active and Healthy Ageing is used because of its increasing importance; and because normal environmental targets are not considered. Results show that; in this case; the most relevant topic to be considered is the primary energy consumption by sources; followed by hazardous solid waste and consumption of non-renewable and scarce materials. According to the experts; these should be the main issues to be considered regarding the environmental sustainability of the outputs of such research and innovation projects. In conclusion; this paper contributes to a better understanding of how to promote a wider integration of environmental sustainability in research and innovation when environmental goals are not initially included.

## 1. Introduction

The worldwide scale and permanent impacts on the planet of human activities which have led to the definition of a new geological period, Anthropocene [1], are well known. Although the new term has not yet substituted the current term Holocene, the suggestion shows the general concern about human participation in shaping the future of the biosphere. This transformation that has lately reached unprecedented levels is being referred to as the Great Acceleration [2,3].

Research and innovation have such a concern as clearly as any other human activity. This is naturally assumed by research and innovation (R&I) actions that aim to support sustainable transitions [4]. However, it could also be assumed by R&I projects focused on other research disciplines and paradigms with potential long-term impacts on the environment.

This is the case of information and communication technologies (ICT) projects for active and healthy aging (AHA). This research has great attention, for instance, in the last European research program Horizon 2020. In the case of the Horizon 2020 program, under the societal challenge “Health, Demographic Change, and Wellbeing”, different calls have been launched to support knowledge production and escalation of ICT-based solutions for active and healthy aging [5,6,7]. The concern on environmental sustainability within these work programs is not a key element and the term sustainability is usually referred to as economic sustainability in regard to the health care system. It is a matter of time before exercises of reflexivity on long-term environmental impacts of the research and innovation outputs will be required by agents submitting proposals to this research field. In the meanwhile, agents within such R&I fields also need to anticipate and manage their present and potential future significant undesirable environmental impacts. However, anticipating the long term environmental impacts involves dealing with a high uncertainty; only increased if applied to research fields not as yet investigated, or driven by research teams not so specifically trained [8].

Therefore, this paper aims to put forward a methodology for actors working at the early stages of R&I projects without environmental goals. The research gap to cover is how to identify the most relevant environmental challenges in order to anticipate the unexpected potential environmental impacts in the medium and long term. To validate it, the ICT projects for AHA are used as a case study.

Next, the literature review about the approaches to the problem is presented; following the methodology that is proposed, the results of its application to the case study, the discussion of results, and the conclusions of the research.

### 1.1. Literature Review

The anticipation of the unexpected environmental impacts of starting R&I is a situation of uncertain and incomplete information [9]. Uncertain and incomplete information refers to the well-known variables of environmental assessment: system life cycle, user habits, the environmental profile of energy in the future, the evolution of materials scarcity, impacts to ecosystems yet to be discovered, etc. The way to react in situations of uncertainty in research and innovation has a long tradition. Different theoretical and conceptual frameworks have been developed in that regard, some of them highlighting the importance of combining expert and non-expert knowledge to deal with uncertainties and advance toward more legitimate responses to global challenges. Concepts such as post-normal science [10], hybrid forums [11], or responsible innovation [12] call for the participation of concerned or interested agents at the early stages of research. Previous research points out that articulating responsible research and innovation systems requires the combination of different strategies and methods, the involvement of different actors [12], and the consideration of context realities [13]. In conclusion, responsible innovation poses a great amount of complexity for R&I practitioners (or policy-makers) and hence, the need for the operationalization of R&I practices, which is the goal of this paper.

### 1.2. Environmental Responsibility of Research and Innovation

The early reflection on environmental sustainability in research and innovation projects could be considered a normative anchor [14], inviting the incorporation of these concerns transversally when designing and thinking about R&I activities and outputs. The various EU directives, policies, commitments, and declarations on the matter justify the need to incorporate environmental concerns about R&I activities. Examples of these normative anchors could be from the Treaty of the European Union until the environmental directives of the DG-EC for Environment, including the Paris Agreement on climate change of 2015, among others. So the considerations on environmental sustainability in R&I projects would respond to the need to take care of the future [12], the expectations of European society [15], and to the proper embedding of scientific and technological advances in society [14].

Based on the framework of responsible research and innovation (RRI) developed by Stilgoe et al. [12], there are two dimensions that could support a better understanding of environmental concerns in early stages of research: anticipation and reflexivity. Anticipation “concerns understanding how the present dynamics of research and innovation practices shape the future and, also, imagining a socially desired future and how to contribute to it” [16]; while reflexivity “ask scientists, in public, to blur the boundary between their role responsibilities and wider, moral responsibilities” [12]. Environmental anticipatory activities in the context of a project would imply to analyze the plausibility of the environmental impacts of the project outputs, or the environmental limitations to scaling up those outputs. The analysis of plausibility done by exploring its possibility, feasibility, and probability [17] would help to foresee environmental conditionings and impacts of the R&I output. Such an understanding of possible environmental implications of a project’s output by the R&I team will activate the reflexivity dimension. The introduction of a new variable in the project design, the environmental responsibility of the project output, is faced with a new moral responsibility. The team needs to position itself (or not) towards a more friendly environmental product. Hence, developing such anticipatory and reflexive exercises during early phases of R&I would support a more conscious approach towards the future. A future steered to some extent by the project’s outputs [18].

With the aim of promoting and monitoring RRI, a group of experts proposed a first attempt at indicators for the policy areas proposed by the European Commission [19]. This framework can inform and support the dimensions suggested by Stilgoe et al. [12]. Nevertheless, neither is the framework applied nor are indicators suggested for the added areas of social justice and sustainability. Some recommendations have been provided in the work of Kettner et al. [20]. However, in their current status, they are more recommendations than practical solutions, and they are more intended for public policies than for environmental assessment. Hence, they would hardly be useful for driving responsible research in practice [21]. 

Technology assessment (TA) is closely related to RRI in aims and approaches. Indeed, Delvenne [22] affirms that the latter appeared in the realm of the former. Since its first appearance around fifty years ago, TA “became a process of ongoing dialogue that supports actors’ decision-making processes and the formation of opinions on science–society issues” [22]. Therefore, this paper research could be said to belong to the overlap between RRI and the branch anticipatory technology assessment [23], with the aim of anticipating and reflecting on the environmental consequences of the R&I process and its outcome, especially if it might turn into a commodity. Currently, anticipatory TA is varied in attitudes and methods, selecting what seems best suitable given the available information and other resources. However, based on the research’s literature review, no proposal including all environmental topics has been found in the realm of anticipatory TA, which could be applied to any type of R&I. Hence, the literature from other disciplines has been reviewed.

The fields of corporate social responsibility (CSR) and sustainable innovation (SI) have devoted more attention to the development of tools covering the anticipation of environmental sustainability [21,24]. Unfortunately, SI is not yet applicable to R&I whose goal is not sustainability [25].

Conversely, in the CSR realm, a variety of guidelines, handbooks, standards, and other tools have been proposed to help integrate the environmental concern in organizations’ operations, even if the environment is not strategic. For a good compendium, see Iatridis and Schroeder [8]. Nevertheless, most of these tools are concerned with the environmental accountability of business rather than with innovation [26,27,28]. Anyhow, the review of CSR literature resulted in a set of potentially useful topics for investigation, as explained in the following section.

Finally, the methodologies of environmental impact assessment (EIA), life cycle assessment (LCA) and strategic environmental assessment (SEA) were also reviewed as potential tools for the goals of the paper. With their differences, all those methodologies were found alien to R&I because of three main reasons [9,29,30]:

- They are developed for concrete projects (EIA), policies/plans/programs (SEA) or product/services (LCA), and are not directly suitable for the ill-definition of the first stages of research and innovation.
- They need to be performed by specialists in environmental assessment, normally not the background of the R&I practitioners this paper addresses.
- They involve a great amount of time, data, and other resources unavailable at the anticipation and reflexivity stages of R&I.

Besides, [9] argued that LCA is not yet effective because its approach is mostly retrospective, when a forward-looking method is necessary. To address this challenge, ex-ante LCA approaches are being proposed [9,31] but they do not yet offer a systematic operationalization for the purpose of our study. Furthermore, although EIA and SEA are different in concept and method, the evidence available suggests that SEA is still largely practiced according to a project’s EIA [32]. This must be the reason why proposals or examples of SEA that could be followed in this paper could not be found.

### 1.3. Environmental Responsibility of ICT for AHA

ICT projects for AHA need digital and electronic devices, i.e., mobile phones, cameras, sensors, senders and receivers, data centres, and servers to process information, etc. The production, use, and disposal of ICT solutions and services may have environmental impacts both at a local and a global level, even if they are deemed less important than other impacts related to security, the privacy of data, or other specific risks. However, ICT for AHA projects are not normally considered to have relevant environmental responsibilities and, thus, they do not normally address environmental impacts among their targets [33,34]. Thus, the case of ICT for AHA involves the use of emerging technologies whose future impacts are too often overlooked [33,35,36]. This is normally due to the lack of awareness, and (or) resources and (or) skills.

This case illustrates the need for the development of context-based approaches that allow the identification of the specific relevance of environmental issues in a specific area of research and innovation. Therefore, the research questions are:

- How to identify the main environmental issues to incorporate them into R&I projects or programs without environmental goals, through anticipation and reflexivity dimensions.
- What those environmental topics might be.
- How to assess the importance of those environmental issues of an R&I project or program in a particular context, e.g., research field.

This paper affirms that anticipation and reflexivity of the environmental impacts is a requirement in R&I projects of ICT for AHA. Europe is aging and a number of ICT projects are being developed in order to improve the elderly’s quality of life [36,37]. However, there is still a research niche in operationalizing this requirement [33]. As an example of the need, some recent studies [38,39,40] conclude that ICT is among the sources relevantly contributing to the increasing levels of CO2 emissions. The idea of predicting possible environmental consequences, especially in the early stages of the project, should, therefore, be a driver for responsible firms or public researchers, interested in the economic benefits and low risks of environmentally sound technologies [33,40,41].

Hence, in this paper we aim to put forward a methodology for identifying, prioritizing, and proposing environmental sustainability elements for anticipation and reflexivity by research groups, not environmental specialists, working in projects not directly related to environmental research [42]. Furthermore, we use the case of ICT for AHA to illustrate its applicability and recommend prioritization of such environmental topics for this specific research field.

## 2. Material and Methods

### 2.1. Methodology

**Figure 1:**
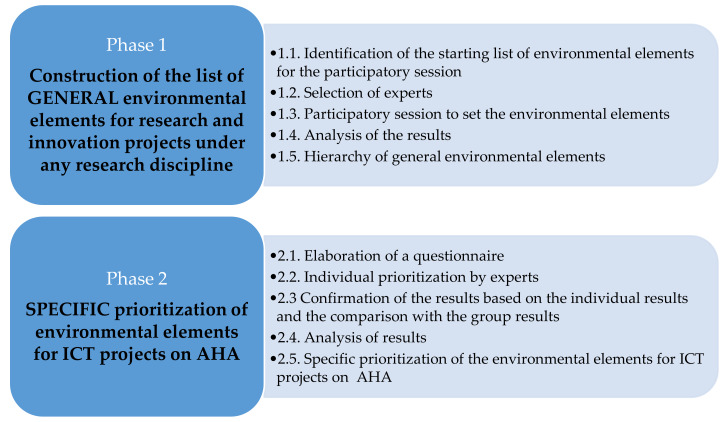
Methodology of the study.

To respond to the objectives of this research we propose a methodology organized in two phases (see Figure 1). The first phase deals with the first research question about which elements related to the environment are to be included in research and innovation projects without initial environmental goals. The set of topics resulting from this first phase is a starting point to design anticipation and reflexivity activities for projects under any line of research and innovation.

The second phase assumes that some of those elements are more relevant for specific lines of research. Therefore, environmental issues are prioritized, illustrating the procedure with the case study of the ICT for AHA.

Therefore, the results obtained in the second phase are valid for articulating anticipation and reflexivity activities for projects of ICT for AHA. The method to obtain the prioritized environmental elements is replicable for other lines of research and innovation.

### 2.2. Methods

The identification of the environmental elements to start with was based on the literature review advanced in Section 1.3. In short, the aim was to identify a guideline or approach that both encompassed all the environmental issues and also had the right level of generality. Moreover, a management approach was desired, as the intention is to help to manage a R&I process in a responsible way.

Participation in the selection of the environmental issues is achieved by means of experts. Those experts will also participate in the rank order of the elements for the case study as AHP is based on expert knowledge and qualitative judgments. Therefore, a group of experts has to be selected with care, and the quality of experts is more important than the number of them, as discussed in García-Melón et al. [43].

In Phase 2, the environmental elements are prioritized by means of the well-known multi-criteria decision-making technique: the analytic hierarchy process (AHP henceforth) [44]. AHP is a measurement theory of intangible criteria based on the fact that the inherent complexity of a multiple criteria evaluation problem can be solved through the construction of hierarchic structures consisting of a goal and several levels of criteria. In each hierarchical level, paired comparisons are made with judgments using numerical values taken from the AHP ratio scale of 1-9. These comparisons lead to dominance matrices from which ratio scales are derived in the form of principal eigenvectors. These matrices are positive and reciprocal (aij = 1/aji). The synthesis of AHP combines multidimensional scales of measurement into a single one-dimensional scale of priorities. These priorities will be calculated for environmental elements.

The AHP method is one of the most extended multi-criteria decision-making techniques (MCDM). In particular, it has been applied in the CSR field [41,43,44] and also to the RRI field [45,46]. Moreover, it has the advantage of being easy to explain to the experts assessing the environmental elements [47]. More details on the AHP can be found in [44,48].

Of all the MCDM techniques, AHP has been chosen because it is the most suitable to work with both quantitative and qualitative criteria. Besides, it is very appropriate when dealing with complex situations with scarce information, such as anticipation of environmental consequences. AHP also helps to manage the consistency of the data, that is, to identify if the experts are inconsistent in eliciting their judgments.

Indeed, many studies have used the AHP to support decision making for environmental assessment, both isolated or connected to other techniques, such as fuzzy theory, the technique for order preference by similarity to ideal solution (TOPSIS), the decision making trial and evaluation laboratory, principal component analysis (PCA), and others [49,50]

Thus, the large number of manuscripts and their wide range of application fields together with the long previous experience of the authors in applying AHP in participatory environments paves the way for its use in this research.

Finally, and no less relevant for this work, the design of an evaluation methodology based on the AHP multi-expert technique allows its replicability. Once the aspects have been defined, their hierarchical structure has been collaboratively constructed and the questionnaires have been created, the technique could be applied in research project scenarios other than the paper’s, by recruiting appropriate experts in the new field of research.

### 2.3. Application of the Method. Phase 1. Construction of the List of General Environmental Elements for Research and Innovation Projects

As stated, the objective of Phase 1 is to propose a list of environmental elements relevant for anticipation in R&I. The list should be holistic, including all the relevant concerns that a research or innovation project might need to anticipate and reflect. For that reason, four activities explained in Figure 1 were carried out, the results of which will be presented in the following section.

#### 2.3.1. Identification of the Starting List of Environmental Elements for the Participatory Session

After the literature review, and aligned with other authors’ proposals [8,27,35], the CSR guidelines and tools were selected for three reasons: (i) they include guidelines and tools with all relevant environmental impacts, (ii) they have a life cycle perspective and (iii) CSR has a management approach, designed to be valid for all sorts of activities, as well as corporate or public research and innovation. Within CSR, the global reporting initiative (GRI) [51] was selected as the source of environmental elements to start the debate among the experts. Various other initiatives and tools were studied and finally discarded for that purpose. To mention the most important ones: ISO 26000 [28], the AA1000 series of standards [52], and the United Nations’ Global Compact [53]. GRI was deemed the most suitable to help to incorporate the full spectrum of environmental questions to all organizations’ activities, regardless of their type, region, or size, based on the dialogue with stakeholders about the materiality of those aspects [8,51]. Besides, GRI presents insights about its monitoring, discussion with stakeholders, and communication, all of which are necessary inputs for later experts’ work in the methodology (see Appendix A and Appendix B). Hence, GRI general environmental indicators act in Phase 1 as the starting information for the discussion about how to anticipate and reflect upon possible future environmental impacts, and to structure the debates among the experts.

#### 2.3.2. Selection of Experts

For the methodology, a participatory approach is proposed as the relevance of the environmental elements is sure to be subject to uncertainty and diversity of preferences. Multi expert participation in such activities is not only crucial for selecting relevant sustainability indicators but also for improving the recognition and use of the indicators [54]. However, it is usually unclear how many participants should be considered in the selection process. Greenbaum [55] proposes that to be considered an appropriate expert for the research, requisites should be: broad experience on the issue, to belong to a specific category of specialists on the problem, and willingness to apply the procedure.

In participatory decision-making procedures based on AHP, the quality of experts is more important than the quantity [56,57]. Ferwati et al. [58] affirm that AHP does not need a big sample size while, after a careful review of the literature, this number was found to greatly vary depending on the type of problem, and the way the model was approached. It is most common to work with a range of 2 to 20 experts. As explained, they are selected because they belong to a certain group or institution [59,60], on the basis of their specific competences in certain fields [61,62], due to their years of experience [63], or for their interest in the problem [64].

In the end, following those rules, we recruited five experts in the field of sustainability, environmental assessment, and environmental education; all of them with professional experience in participating and managing R&I projects, and with different professional roles. The experts were selected because they were capable of applying their knowledge in the first phase to provide a general list of environmental topics for projects under any research line. And in the second phase, because they could contribute to identifying those criteria which were more relevant and urgent in the field of ICT for AHA. Table 1 presents the different experts’ profiles.

#### 2.3.3. Participatory Session to Set the Environmental Elements

A meeting with the experts was arranged. They met in June 2018. First, they reviewed and accepted the proposal of the list based on the GRI universal environmental indicators. During the meeting, they analyzed the environmental topics proposed in the GRI indicators. Then, the description of the GRI environmental indicators was used to structure the debate among the experts. A deductive analysis allowed discussion of the validity of the GRI indicators and the identification of new environmental general elements. This resulted in a new set of elements aligned with the specificities of research and innovation projects.

#### 2.3.4. Analysis of the Results

After the participatory session and considering the discussions among the experts, a definition of each element on the list was proposed. In addition, the elements were hierarchized to group them in categories and allow prioritization in the second phase. The experts received the definitions and the hierarchy to confirm that they respected the agreements of the participatory session (see the section of Results and Appendix B for the hierarchy and description of each element). Thus, the hierarchy can be used as a complete list of environmental issues to discuss during the anticipation and reflexivity activities of any R&I project. These results are presented and commented upon in the sections of Results and Discussion.

### 2.4. Phase 2: Prioritization of the Agreed Environmental Issues for ICT Projects on AHA

The objective of phase 2 is to propose a methodology for identifying the most relevant topics for a specific research line in a way that a tailored, reduced set of elements can be provided. Built upon the hypothesis that there are environmental elements that are more important to consider in certain projects, the aim is to avoid overburdening researchers by discarding those with lesser impact in their projects.

In this phase, the experts were asked to prioritize the environmental elements for projects of ICT for AHA. As described in the introduction, ICT for AHA projects do not usually focus on reducing the environmental impact of the outputs of the research. Hence, experts explored the connections of ICT for AHA and environmental sustainability. The prioritization phase required the completion of the following tasks.

#### 2.4.1. Elaboration of a Questionnaire

A questionnaire was developed, with the list of environmental elements resulting from the first phase. The questionnaire allowed the experts to compare two elements of the same level of the hierarchy following the AHP method. The questionnaire included two examples of these types of projects to ensure that the experts were acting bearing in mind the same type of research disciplines. Those two examples were based on real projects funded under the Horizon 2020 research program of the European Commission (FrailSafe and Activage project). An example of a section of the questionnaire is included in Table 2.

The used questionnaire is the standard questionnaire for paired comparisons required by the AHP matrices. Comparisons between criteria are made pairwise. The questionnaire uses the Saaty fundamental scale [44], which is a 9-point ratio type scale, where 1 means equally important and 9 extremely more important. In this example, as number five was highlighted, the asked expert judges the cluster *Flows from biosphere* much more relevant than the cluster *Flows to biosphere*, in order to anticipate and reflect on the environmental impacts of ICT projects applied to AHA.

As the experts need not be familiar with the questionnaires, each time an expert was asked, the AHP facilitators accompanied them during the task, helping to sort out the difficulties. Besides, AHP allows the identifying of inconsistencies in the experts’ judgments that, when they appeared, were also discussed and solved with the aforesaid experts.

#### 2.4.2. Prioritization of ICT for AHA by the Experts

The questionnaires were issued out to each expert and they answered according to their level of preference following the Saaty 1–9 fundamental ratio scale. After processing the individual responses using Superdecisions^®^ software, the individual and the whole group results were compiled.

This approach ensured that the prioritization of the environmental elements was ICT-specific and based on the environmental experts’ perception of the current relevant topics on this line of research. Therefore, the participatory procedure resulted in a set of prioritized environmental elements that are research-area specific.

#### 2.4.3. Confirmation of the Results based on the Individual Results and Comparison with the Group Results

After obtaining the results derived from the analysis of the questionnaires, both the individual results of each participant and the group results were sent to each expert so that they could confirm them or, otherwise, modify any of their individual judgments. Two experts expressed their aim to adjust their judgments and did so.

#### 2.4.4. Analysis of the Results

After the revisions, the questionnaires with the final judgments of the experts were analyzed with superdecisions. As proposed by Saaty and Peniwati [57], the aggregation of all the individual judgments was calculated by means of the geometric mean to obtain the prioritization by the group of experts. The results of the phase two are presented and commented upon in the next section.

## 3. Results

The results of the study can be grouped into two categories. On the one hand, the first phase resulted in a panel of environmental elements organized in a hierarchy. The hierarchy included all the topics that the experts considered should be used in any research and innovation project without initial environmental goals, as a starting point to design the content of anticipation and reflexivity activities. On the other hand, as a result of the second phase, a prioritization is obtained of the environmental elements for a specific line of research, ICT for AHA.

### 3.1. Results from the First Phase: Hierarchy of Environmental Elements for Anticipation and Reflexivity Activities for any Research Line

**Figure 2:**
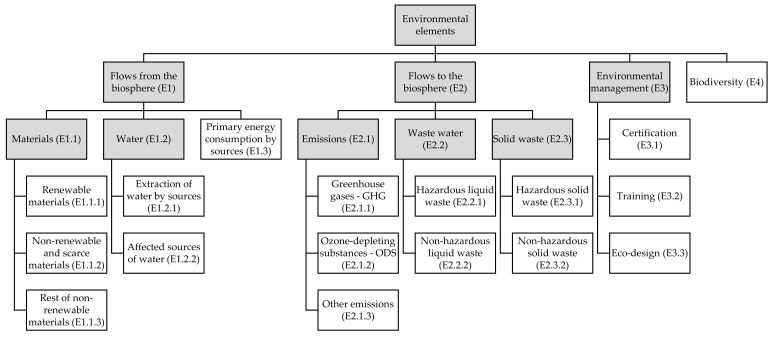
Hierarchy of the environmental elements for anticipation and reflexivity activities for any research line without initial environmental goals.

The hierarchy obtained included twenty-five environmental elements and is presented in Figure 2. As can be seen, the environmental elements have been arranged in clusters by the experts. To achieve that, the general guidelines of GRI were debated and, applying a simple tree-building technique, the elements were classified in levels of specificity, and grouped by similar environmental features: Flows to Biosphere, Flows from Biospheres, etc. For definitions of each element, see Appendix B. This hierarchy contained the environmental issues that research and innovation projects of any topic should use to design anticipation and reflexivity activities on the potential intended and unintended consequences of the outputs of their projects and the scaling up of those products.

The hierarchy is based on the GRI proposal, and the adaptation to the R&I activity carried out by the experts. Thus, it is a list of elements closely related to the environmental consequences of R&I, the kind of information its stakeholders may demand, and the elements can readily be turned into indicators for monitoring, management, and disclosure if need be. Hence, these elements help to operationalize the dimensions of reflexivity and anticipation in line with other proposals such as [8,16,65,66]

This hierarchy aims to be complete more than to be usable, i.e., the model will normally be too complex for the anticipation and reflexivity of an R&I project. Therefore, for its application, specific to a research area, the hierarchy has to be prioritized and the relevant environmental elements be distinguished from the rest. Hence, the need for the second phase in the procedure, the one illustrated with a case study in the next section.

### 3.2. Results from the Second Phase: Prioritization of Environmental Elements for ICT for AHA

The second phase of the study resulted in a prioritization of the environmental elements of the hierarchy obtained from phase 1. This prioritization was tailored for projects dealing with ICT for AHA solutions. The prioritization presents the order and percentage of importance that the experts assigned to the seventeen environmental elements at the end level of the hierarchy (with no sub-elements) for the specific case of ICT for AHA research and innovation projects.

The ranked environmental elements are presented in Table 3. This table shows in rows the elements ordered by importance (last column for the group), and in columns, the percentage assigned to each of them by the individual experts, together with the group aggregation.

These data show that in a given group of experts such as the ones participating in this study, there are different perceptions about the importance of the compared elements in order to reach a specific objective (e.g., anticipate and reflect on relevant environmental impacts of ICT on AHA projects).

**Figure 3:**
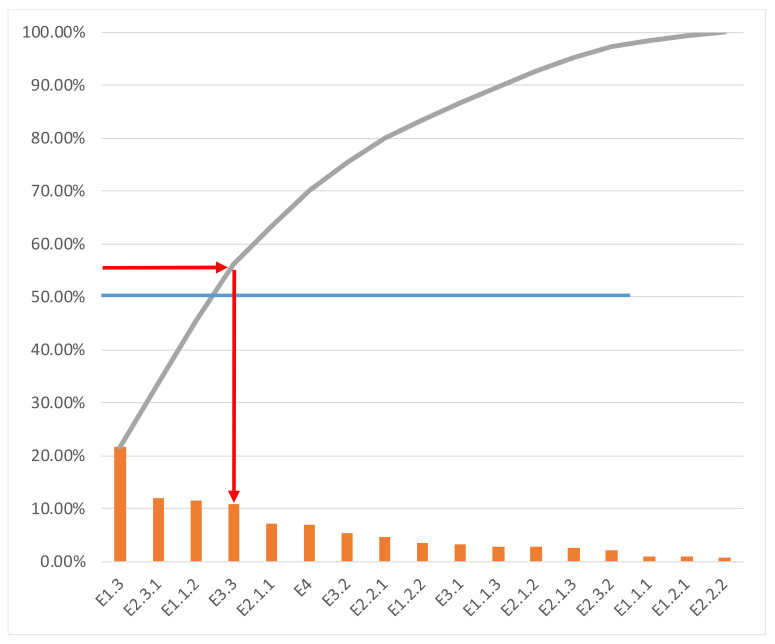
Application of the criterion “up to 50%” to select the most relevant environmental elements according to the group of experts.

This ranking is useful to identify the most relevant elements, by agreement or average, to be considered in the design of anticipation and reflexivity activities related to environmental sustainability. In order to use this result in the design of such activities, it might be useful to choose the most representative elements. In previous studies by the authors, this has been done by identifying the elements of the list that represent 50% of the total weight [65]. The application of this criterion will produce a tailored-reduced panel of elements for ICT for AHA projects, resulting in a more manageable list of topics in cases of the scarcity of resources such as specific knowledge and time. Figure 3 shows this procedure. As can be seen, the weight of each criterion is displayed as a bar, and the curve line shows the accumulated weight after adding each element’s weight. The first four elements altogether represent 56.07% of the total weight.

## 4. Discussion

Normally, ICT research is much more concerned with goals such as the process speed, reliable communication, wide-coverage, data privacy, duration of service, etc. In fact, ICT for AHA, in the same way as many other western paradigms, is rooted in a socio-political framework of growth based-economies [67]. Reducing the environmental burden on this line of research might imply swift forward strategies aligned with the principles of de-growth, which might not be naturally considered without an explicit exercise to anticipate and reflect on the long-term impacts of this research line outputs. The use of the proposed relevant environmental elements for a specific R&I domain could be used to inform through the design of the several activities and strategies identified to be contributing to these dimensions [41].

Comparing the results with the literature on ICT and sustainability, for example [18,39,40], there are clear coincidences with the outcomes of those research teams. Elements such as primary energy consumption, hazardous waste, greenhouse gas emissions, and consumption of scarce materials are the top concerns for the life cycle of ICT technologies. Or to give another example, eco-design and eco-innovation are consistently mentioned as in need of more attention in ICT development [38,68]. Thus, these results and methodology contribute to raising awareness on the most relevant environmental issues of any research without environmental goals. Furthermore, in order to feasibly integrate the environmental concerns during the R&I practice, a methodology has been applied to focus on the most relevant elements. In the case of ICT for AHA, as demanded by [34,39], it operationalizes and informs the critical first steps of the anticipation and reflexivity on the future environmental impacts of these R&I projects.

Regarding the methodology, AHP has proven to be a convenient tool to model a decision-making problem in which data are not complete, i.e., the expected environmental impacts and the available data are often qualitative and/or uncertain. AHP is based on experts that have a clear enough idea of how to compare environmental elements for anticipation and reflexivity during research and innovation. The results of the method were deemed by the experts to represent what they know, to convey their experience on the environmental assessment of R&I, and particularly, on the case study of ICT for AHA.

As introduced, the results in Table 3 show that there are significant discrepancies among the experts, although there is an overall agreement about the relevance of most elements. For example, ‘(E1.3) Primary Energy Consumption’ of the product-to-be has been ranked as the most important element for anticipation and reflexivity by the group, also by experts 3, 4, and 5, and the second most relevant by expert 2. However, for expert 1 it is not even relevant, and she maintained her opinion after knowing the other experts’ preferences.

Another example of discrepancies is the element ‘(E3.2) Training’. It is the most relevant element for expert 1 (together with ‘Eco-design’) and expert 2 (clearly differentiated from the rest). However, it has so little importance to experts 3, 4, and 5 that, for the group, it is only the 7th in weight of the selected elements. This situation is normal when discussing the importance of environmental concerns from different approaches. Decision-makers, in this case, R&I practitioners, have to finally align with some experts or others, or with an average preference, aggregating all judgments.

AHP can also help the discussion as it shows the individual and the aggregated preferences, and it is fully disclosed and traceable. Specific judgments (pairwise comparisons) leading to the elements’ preferences can both be acknowledged and discussed. Afterwards, a cut off rule can be applied to trim the list of elements to be assessed.

In this case study, the weights of the aggregated model do not differ much and the cut off was set at 50%. A trade-off is necessary between including as much importance (weight) as possible and keeping the list of elements simple. However, it is debatable where this threshold should be fixed.

Considering the experts’ profiles, experts 3, 4, and 5 who work at universities coincide clearly. On the other hand, expert 1 (at a business association) and expert 2 (at a research center) show different preferences from the academics, and also between them.

Finally, whenever an interview with experts takes place, there will always be comments apart from the questionnaire that enrich the results. In this case, some of the insights provided by the experts were:

i) The selection of elements for reflexivity/anticipation could vary somehow from one region to another, for example, as the primary energy mix may be more or less polluting in different countries. This also applies to the evolution with time. It is expected that electricity will become ever less polluting in the industrialized countries, while breakthrough innovations may solve the problem of e-waste, scarce materials, etc.
ii) Given the topics, throughout an R&I project, it is not clear if it would be more convenient for the R&I team to get training in those environmental matters, to incorporate environmental experts to the project, or to add them to the stakeholders to achieve dialogue.

Hence, this work must be reviewed periodically, updating the list of environmental elements, and their preferences for particular research fields, like ICT for AHA. Additionally, the results of this research are somehow biased by the region where experts live, mainly Spain. Thus, the prioritization of environmental elements for particular case studies will not only consider the features of these cases but, also, will be influenced by the region where those projects will be carried out. For example, it is not the same to design devices that will consume currently polluting Spanish electricity than the much cleaner current Finnish electricity. Furthermore, research work such as [33,39,40] add insights on the advantages and disadvantages of either incorporating environmental experts to the project or to outsource that part.

The study focused on anticipation and reflexivity regarding the outputs of research and innovation activities. Other studies on R&I have pointed out the importance of intervening at the dimensions of “Process” and “Perception” in the stakeholders’ network to reach the objectives of this policy [19], which have not been an object of this study.

## 5. Conclusions

In a situation of an ever-increasing application of ICT tools for active and healthy aging, their environmental impacts, however small, will be relevant by accumulation. Hence, the ever-increasing demand for environmental responsibility will reach R&I projects without initial environmental goals. Hence, this research is framed in the approach around the concept of responsible innovation and, specifically, in the need for anticipation and reflexivity of potential impacts of research and innovation to respond to the demands of society. The research contributes to operationalize this response in R&I without environmental goals. This model of the environmental concerns of R&I practice helps to raise awareness and to identify the problems to anticipate and reflect about. While the method for ranking ordering the environmental concerns of specific research fields enables the feasibility of the task.

As the environmental consequences of R&I outcomes are a wide and complex problem, GRI has been applied to divide them into clusters, elements, and their connections and hierarchy. Then, each element can be dealt with separately, though considering its role in the whole model.

To achieve it, the knowledge of five experts is processed. Experts on research and innovation, environmental assessment, and, to a lesser extent, ICT for AHA. Their job was to identify the environmental elements and to rank order them to enable an effective and efficient carrying out of the anticipation and reflexivity tasks.

However, this proposal has its limitations. On the one hand, the outcomes are temporary and must be updated as the understanding of the environmental causes and consequences, and the main challenges of each period evolve. On the other hand, while the hierarchy of environmental elements is quite consistent with the literature, and thus a good manageable summary, the rank order of the elements is case-specific, and very debatable. Indeed, the consensus among experts was impossible in this case. This situation is frequent and does not invalidate the procedure. It reflects the aforementioned uncertainty about future environmental impacts. Hence, it has the positive effect of informing R&I practitioners on the intrinsic difficulties of anticipation and reflexivity and the possible debates. Furthermore, it also gives key concepts and arguments for a realistic balance among environmental goals and other R&I project goals. Finally, it is still the R&I team’s task to engage stakeholders in a debate on the elements, contributing to what has been defined as forward-looking moral responsibility. The approach has to be pragmatic because better monitoring is achieved (i.e., better data, a better understanding of the data, more appropriate recommendations, and better uptake of findings); but also ethical, because it is the right thing to do (i.e., people have a right to be involved in informing the decision making process, whose outcomes will directly or indirectly affect them). Stakeholder participation is crucial also for improving the recognition and use of the reflexivity results, and to contribute to the consideration of a shared-responsibility.

Finally, the prioritized elements could form part of training contents to increase research and innovation teams’ capabilities to enhance sustainable innovations. They could also serve to focus the elements to be reviewed in interdisciplinary collaborations. It can be argued that the integration of such exercises in the ICT for the AHA domain could lead to a better understanding of how to reduce potential unintended environmental impacts of massive promotion of technologies for supporting active and healthy aging. Anticipation and reflexivity might commit researchers and innovators in imagining more environmentally respectful technologies and to reflect on the norms and assumptions behind the development of their research outputs. Creative solutions and new imaginaries might appear to tackle the challenge of an aging population by focusing on research and innovation efforts based on ICT in reducing the environmental burden of their massive application in Europe.

## Figures and Tables

**Table 1:** Experts’ profile.

Expert	Profile
Expert 1	Senior researcher expert in life cycle ssessment, with responsibility in the environmental part of national and European research and innovation projects.
Expert 2	Coordinator of environmental educational activities and project manager of European projects.
Expert 3	Professor, specialist in life cycle assessment, main researcher of various national and European projects.
Expert 4	Professor with experience as an evaluator of research and innovation projects. Expert in environmental assessment.
Expert 5	Professor and expert on pollution prevention and control.

**Table 2:** Experts’ profile.

From Your Point of View, Which Element is More Important, and to What Degree Does It Anticipate/Reflect on the Environmental Impacts of ICT Projects Applied to AHA?										
E1. Flows from biosphere	**9**	**7**	**5**	**3**	**1**	**3**	**5**	**7**	**9**	E2. Flows to biosphere

**Table 3:** Prioritized list of environmental elements for anticipation and reflexivity activities for ICT for AHA projects. Individual weights assigned by each expert (E) in percentage, and aggregated weight for the group.

Environmental Element	E1	E2	E3	E4	E5	Aggregated (Group)
Primary energy consumption by sources (E1.3)	3.0%	17.1%	21.5%	36.3%	27.9%	21.54%
Hazardous solid waste (E2.3.1)	2.5%	9.7%	9.6%	16.9%	8.4%	12.06%
Non-renewable and scarce materials (E1.1.2)	6.5%	4.5%	1.6%	11.7%	9.7%	11.61%
Eco-design (E3.3)	22.7%	4.5%	7.8%	9.2%	2.9%	10.86%
Greenhouse gases GHG (E2.1.1)	3.8%	0.8%	15.0%	3.8%	5.4%	7.0%
Biodiversity (E4)	9.6%	3.9%	7.0%	3.9%	8.0%	6.7%
Training (E3.2.)	22.7%	38.5%	1.2%	0.8%	0.8%	5.38%
Hazardous liquid waste (E2.2.1)	7.4%	3.0%	2.8%	1.5%	2.6%	4.52%
Affected sources of water (E1.2.2)	7.8%	1.1%	1.7%	2.9%	2.3%	3.49%
Certification (E3.1)	3.2%	1.2%	2.4%	1.7%	0.3%	3.32%
Rest of non-renewable materials (E1.1.3)	2.0%	0.4%	4.6%	3.3%	3.0%	2.90%
Ozone-depleting substances ODS (E2.1.2)	1.3%	0.1%	6.5%	0.6%	23.2%	2.90%
Other emissions (E2.1.3)	3.8%	0.2%	1.7%	1.5%	2.9%	2.58%
Non-hazardous solid waste (E2.3.2)	0.5%	1.4%	1.6%	4.2%	1.2%	2.13%
Renewable materials (E1.1.1)	0.5%	0.9%	1.0%	0.9%	0.7%	1.04%
Extraction of water by sources (E1.2.1)	1.1%	0.4%	1.7%	0.6%	0.5%	1.01%
Non-hazardous liquid waste (E2.2.2)	1.5%	0.4%	0.4%	0.2%	0.4%	0.69%
Total	100%	100%	100%	100%	100%	100%

## Abbreviations

AHA—Active and Healthy Ageing

AHP—Analytic Hierarchy Process

ICT—Information and Communication Technologies

RI—Responsible innovation

R&I—Research and Innovation

RRI—Responsible Research and Innovation

TA—Technology Assessment

## Appendix A

**Table A1:** Indicators of the GRI Environmental Standards G4–300 series.

Environmental Topic	Indicators
GRI 301: Materials	301-1 Materials used by weight or volume 301-2 Recycled input materials used 301-3 Reclaimed products and their packaging materials
GRI 302: Energy	302-1 Energy consumption within the organization 302-2 Energy consumption outside of the organization 302-3 Energy intensity 302-4 Reduction of energy consumption 302-5 Reductions in energy requirements of products and services
GRI 303: Water and Effluents	303-1 Interactions with water as a shared resource303-2 Management of water discharge-related impacts 303-3 Water withdrawal 303-4 Water discharge 303-5 Water consumption
GRI 304: Biodiversity	304-1 Operational sites owned, leased, managed in, or adjacent to, protected areas and areas of high biodiversity value outside protected areas 304-2 Significant impacts of activities, products, and services on biodiversity 304-3 Habitats protected or restored 304-4 IUCN Red List species and national conservation list species with habitats in areas affected by operations
GRI 305: Emissions	305-1 Direct (Scope 1) GHG emissions 305-2 Energy indirect (Scope 2) GHG emissions 305-3 Other indirect (Scope 3) GHG emissions 305-4 GHG emissions intensity 305-5 Reduction of GHG emissions 305-6 Emissions of ozone-depleting substances (ODS) 305-7 Nitrogen oxides (NOX), sulfur oxides (SOX), and other significant air emissions
GRI 306: Effluents and Waste	306-1 Water discharge by quality and destination 306-2 Waste by type and disposal method 306-3 Significant spills 306-4 Transport of hazardous waste 306-5 Water bodies affected by water discharges and/or runoff
GRI 307: Environmental Compliance	307-1 Non-compliance with environmental laws and regulations
GRI 308: Supplier Environmental Assessment	308-1 New suppliers that were screened using environmental criteria 308-2 Negative environmental impacts in the supply chain and actions taken

## Appendix B

**Table A2:** Description of the elements of the first level.

Elements of First Level and Code	Description
Flows from the biosphere (E1)	This element includes the elements related to the extraction of existing resources in the biosphere: raw materials, energy, and water mainly. These extracted resources alter the composition of the biosphere and its ecosystem equilibria and limit their availability for future generations.
Flows to the biosphere (E2)	This element groups the substances that are released into the biosphere, altering their composition and their eco-systemic equilibria.
Environmental management (E3)	This element groups the elements of reflexivity on what the research team can do in relation to the protection of the environment.
Biodiversity (E4)	This element addresses those elements related to reflexivity on how the research, or the product of it, can directly impact the species in danger of extinction, or their habitats.

**Table A3:** Description of the elements of the second level.

Element of First Level	Element of Second Level	Description
Flows from the biosphere (E1) ^1^	Materials (E1.1)	This element groups everything related to the different materials that will be consumed, renewable, non-renewable, etc.
	Water (E1.2)	This element includes both the extraction of the different types of water throughout the R & D & I project and the life cycle of the products derived from the research, as well as the consequences that these extractions have on water sources.
	Energy (E1.3)	This element addresses the extraction of energy resources, that is, the consumption of primary energy by sources, for the entire project and the life cycle of the research products
Flows to the biosphere (E2) ^2^	Emissions (E2.1)	This element groups everything related to the different gaseous emissions of substances that will be produced during the R & D & I project and during the life cycle of the product resulting from the investigation.
	Wastewater (E2.2)	This element groups the liquid discharges with polluting load that will be carried out during the project and during the life of the product of the investigation.
	Solid waste (E2.3)	This element groups the solid waste with environmental impact for the entire project and the life cycle of the research products.
Environmental management (E3) ^3^	Certification (E3.1)	This element addresses all the activities that the research team can carry out aimed at verifying and, where appropriate, certifying that the actions, the suppliers of goods and services, the facilities and equipment, the products of the research, etc. comply with environmental requirements. Requirements that are normally more demanding than legislation, although not always
	Training (E3.2)	This element addresses the team’s activities aimed at improving awareness and competence in the protection of the environment during the project and during the life of the product developed. It refers to the awareness and competence of researchers and directly related stakeholders: research partners, suppliers, beneficiaries, funders, etc.
	Eco-design (E3.3)	This element addresses the activities aimed at changing the design of research and research products so that environmental impacts are reduced throughout their life cycle.

^1^ The experts who selected the elements of reflexivity specified that the research or innovation team should assume the possible responsibility for these energy consumptions with a perspective of the life cycle of the project and its possible product. Also that direct and indirect consumption should be considered, and that relative consumption should be estimated, by functional unit of the project and the product, in contrast with an absolute estimate of total numbers. “Functional unit”, according to the definition of the UNE-EN ISO 14040: 2006 standard, refers to the "Quantified performance of the product system for use as a reference unit" and "Product", according to the same norm, refers to tangible objects, but also to research services in an R&I project. ^2^ It has been decided to separate the waste that is generated in a waste discharge that is generated in solid state. Although all should be managed properly, in reality there are leaks and bad practices, and it has been considered that it is not the same when this happens with a solid or semi-solid waste, compared to a totally liquid one. Regardless of the origin of the waste and in what medium that residue ends. ^3^ These three elements have strong connections with each other but should be considered as isolated. It is a forced independence, but not impossible. We want to evaluate what is considered to be the most influential in the environmental responsibility of the research team, for ICT projects. In addition, the experts agreed that the research teams should study how some actions or others contribute to their environmental responsibility from the point of view of the direct impact on the research and its product, but without taking into account indirect effects.

**Table A4:** Description of the elements of the third level.

Elements of Second Level	Elements of Third Level	Description
Materials (E1.1) ^1^	Renewable materials (E1.1.1)	This element refers to all the materials that the biosphere renews on a time scale compared to the human scale. Basically, they are the primary organic materials (not cultivated) such as wood, fish, guano and other natural fertilizers, etc. that would be used during the research project and during the life cycle of the research product
	Non-renewable and scarce materials (E1.1.2)	This element refers to the consumption of different scarce materials that the biosphere may never renew or will employ a time scale much greater than the human scale. Minerals such as Coltan, Titanium, etc. are included. But fossil fuels are not included, they go in the element E1.3.
	Rest of non-renewable materials (E1.1.3)	This element refers to the consumption of different materials that, like Silicon, Lithium, Iron or others, are very abundant at present, but as they are not renewable, their availability decreases, apart from the impact that their extraction has on habitats
Water (E1.2)	Extraction of water from sources (E1.2.1)	This element addresses all the water extractions that are carried out during the project and the life of the product. Likewise, the experts who selected the elements of reflexivity specified that the team should assume the possible responsibility for this water consumption with a perspective of the life cycle of the project and its possible product. Also, that direct and indirect consumption should be included, and that relative consumption should be estimated, by the functional unit of the project and the product, in contrast to an absolute estimate of total numbers.
	Affected sources of water (E1.2.2)	This element addresses all water sources that have reduced their contribution, or worsened their quality, or suffered any other environmental impact. The experts who selected this element specified that the team reflected on the number of directly affected sources, and the intensity of the effect, per functional unit, with a life cycle perspective of the project and its possible outcome.
Emissions (E2.1)	Green-house gases–GHG (E2.1.1)	This element addresses all the greenhouse gas emissions that are made during the project and the life of the product. For example, methane, carbon dioxide, dinitrogen monoxide, etc. The experts indicated that the team should assume the possible responsibility for these direct and indirect emissions, with a perspective of the life cycle and functional unit of project and product of the project.
	Ozone-depleting substances–ODS (E2.1.2)	This element addresses all gaseous emissions of substances that attack stratospheric ozone: CFCs, HCFCs, etc. They already occur directly or indirectly, by the functional unit, and with a perspective of the life cycle of the project and its possible product.
	Other emissions (E2.1.3)	This element addresses the gaseous emissions of other polluting substances that are directly, but not indirectly, emitted per functional unit during the life cycle of the project and its possible product. They include, for example, solid particles in suspension, volatile organic compounds, sulfur oxides, etc.
Wastewater (E2.2)	Hazardous liquid waste (E2.2.1)	This element includes all those that can be given in the liquid form and which, due to their composition and origin, are classified as hazardous according to the European Waste List. The experts indicated that the team should assume the possible responsibility for these direct and indirect discharges, with a perspective of the life cycle and functional unit of project and product of the investigation.
	Non-hazardous liquid waste (E2.2.2)	This element includes all those not included in the European List, which occur directly, but not indirectly, bythe functional unit, and with a perspective of the life cycle of the project and its possible product.
Solid waste (E2.3)	Hazardous solid waste (E2.3.1)	This element includes all those that may occur in solid or semi-solid state and that, due to their composition and origin, are classified as hazardous according to the European Waste List. The experts indicated that the team should assume the possible responsibility for these direct residues, but not indirect ones, with a perspective of the life cycle and functional unit of project and product of the investigation.
	Non-hazardous solid waste (E2.3.2)	This element includes all those not included in the European List, which occur directly, but not indirectly, by the functional unit, and with a perspective of the life cycle of the project and its possible product.

^1^ The element E1.1. “Materials” can be divided into three elements of the third level. It is important to highlight that the experts who selected the elements of reflexivity specified that the team should assume the possible responsibility for these material consumptions with a perspective of the life cycle of the project and its possible product. Also that direct and indirect consumption should be included, and that relative consumption should be estimated, by functional unit of the project and the product, in contrast to an absolute estimate of total numbers.
